# Lifetime and acute risk of suicidality by profiles of early life adversity: an observational cohort study in a high-risk population

**DOI:** 10.1038/s41598-026-60185-7

**Published:** 2026-07-01

**Authors:** Johannes Wolf, Stephan Goerigk, Gerrit Burkhardt, Julia Eder, Julia I. Kunz, Peter Falkai, Katja Bertsch, Thomas Ehring, Andrea Jobst, Matthias A. Reinhard, Frank Padberg

**Affiliations:** 1 Department of Psychiatry and Psychotherapy, LMU University Hospital, LMU Munich, Nussbaumstr. 7, 80336 Munich, Germany; 2 German Center for Mental Health (DZPG), Partner Site Munich-Augsburg, Munich, Germany; 3 Charlotte Fresenius Hochschule, Munich, Germany; 4 Department of Psychology, Julius-Maximilians-Universität, Würzburg, Germany; 5 Department of Psychology, Ludwig-Maximilians-Universität, Munich, Germany

**Keywords:** Diseases, Health care, Medical research, Psychology, Psychology, Risk factors

## Abstract

Early life adversity in the form of child maltreatment (CM) is a transdiagnostic risk factor for suicidal ideation and behavior (SIB), yet its heterogeneity and relevance for lifetime versus acute SIB and psychotherapy outcomes remain unclear. In an observational cohort of 240 high-risk adults with borderline personality disorder (42.5%) or persistent depressive disorder (57.5%), lifetime and acute SIB were assessed using the Columbia–Suicide Severity Rating Scale. CM profiles were derived using a data-driven clustering approach based on the Childhood Trauma Questionnaire. Associations between CM profiles, SIB, and symptom change during inpatient cognitive behavioral analysis system of psychotherapy (CBASP) or dialectical behavior therapy (DBT) were examined. Seven distinct CM profiles were identified. A profile characterized by physical and emotional abuse and emotional neglect was associated with elevated lifetime risk for active suicidal ideation with plan and intent and for potentially lethal suicide attempts, compared with minimal CM. CM profiles were not associated with acute SIB or their reduction during inpatient psychotherapy, although differential depressive symptom change by CM profile was observed following CBASP but not DBT. These findings suggest that CM profiling may inform lifetime SIB risk assessment and require replication in larger samples.

**Supplementary Information:**

The online version contains supplementary material available at 10.1038/s41598-026-60185-7.

## Introduction

Suicide is a leading cause of mortality worldwide, and suicide rates remain high despite the identification of numerous risk factors and the development of specific psychological interventions^1–3^. Suicidal ideations and behaviors (SIB) are particularly pervasive among people living with severe mental health disorders, such as individuals with borderline personality disorder (BPD) and chronic depression, i.e. persistent depressive disorder (PDD) according to DSM-5^2,4–6^. The differential evaluation of SIB from passive ideation to potentially lethal suicide attempts and from lifetime to acute risks and risk factors relating to these different forms of SIB is particularly important to inform mental health care routines for these clinical groups. Analyzing the differential response to psychological interventions in SIB high-risk subgroups may help to effectively tailor interventions for people with SIB^7–10^.

Early life adversity, specifically child maltreatment (CM), was shown to be a transdiagnostic risk factor for SIB^11,12^. Several subtypes of CM contribute to the overall CM burden. The most commonly reported CM subtypes include experiences of neglect, emotional abuse, physical abuse, and sexual abuse. These subtypes have been shown to contribute individually to an increased suicide risk^11^, with recall of childhood physical abuse being associated with the highest risk for SIB in people with BPD and PDD^6^. However, patients rarely report single subtypes of CM. In contrast, many studies showed that CM subtypes co-occur in complex patterns and vary considerably in their severity^13–18^. Moreover, more severe CM burden was associated with more severe psychopathology^19,20^. To adequately capture the complexity of adversity in early family environments as a risk factor for SIB, a novel approach based on the Childhood Trauma Questionnaire (CTQ) identified distinct CM profiles representing patterns of co-occurrent CM subtypes and their severity according to individual recall^21^. This CM profiling approach was more informative regarding depressive symptoms and loneliness than psychiatric diagnoses in a transdiagnostic sample^21^ and differentially predicted individual responses to a disorder-specific psychotherapy (i.e., cognitive behavioral analysis system of psychotherapy; CBASP)^22^. To date, this approach has been replicated by our group^21,22^ and beyond^23^ but has not been applied in the context of SIB.

This study aimed to apply the CM profiling approach to stratify SIB risk from a lifetime versus acute perspective and assess change in acute SIB and disorder-specific symptoms during inpatient psychotherapy programs in a cross-diagnostic sample of adults with BPD and PDD. Both groups were chosen since they showed high levels of SIB and CM in previous studies and the transdiagnostic research line of our group has a particular focus on these groups^4,6,11,24–27^. Our main hypotheses were that participants within different CM clusters display different levels of lifetime and acute SIB. Our secondary hypotheses were that individuals in different CM clusters show a differential change after inpatient psychotherapy programs in terms of acute SIB and disorder-specific symptoms (depressive or borderline symptoms respectively).

## Results

### Demographic characteristics of the sample

Complete data of 240 adults (160 [66.6%] women; mean age: 32.1 years [SD 12.1]) were available for analyses: 102 (42.5%) individuals with BPD were treated with DBT and 138 (57.5%) individuals with PDD were treated with CBASP. Sample comorbidity is reported in the Supplement (see Supplementary Table 1). In the total sample, comorbid affective disorders (75.4%), anxiety disorders (50.4%), and personality disorders (40.8%) were most common.

### Child maltreatment profiles derived from the dimensions of the childhood trauma questionnaire

**Fig. 1:**
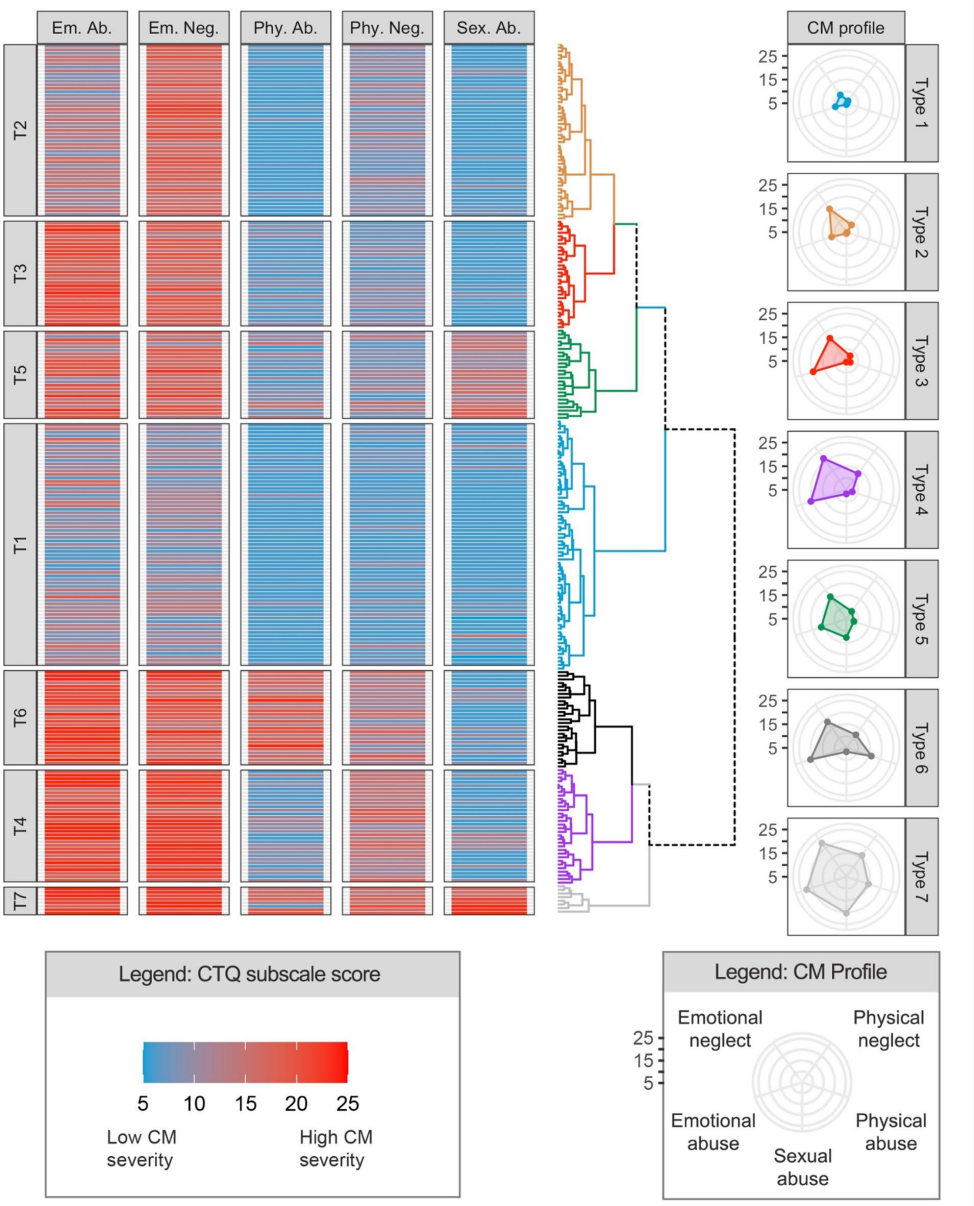
Derivation of clusters from the dimensions of the Childhood Trauma Questionnaire. The heatmap illustrates the self-reported severity of CTQ dimensions for individual patients, with colors transitioning from blue to red indicating a shift from low to high levels of child maltreatment severity. CTQ scores served as the basis for defining clusters of child maltreatment, which were identified through agglomerative hierarchical clustering. CTQ patterns, labeled types 1–7, are distinguished by varying combinations of maltreatment dimensions.

Seven CM profiles were identified with distinct combinations of CM patterns and severity. Cluster type 1 represented no CM (69 [28.7%] patients); type 2 represented minimal CM (49 [20.4%]); type 3 showed predominant emotional abuse (30 [12.5%]); type 4 indicated predominant emotional neglect and emotional abuse (32 [13.3%]); type 5 revealed predominant emotional neglect, emotional abuse and some experiences of sexual abuse (25 [10.4%]); type 6 represented emotional neglect, emotional and physical abuse (27 [11.2%]); and type 7 reported emotional neglect, emotional and sexual abuse (8 [3.3%]). Sample characteristics of each profile are summarized in Table 1. A visual representation of the CM profiles is depicted in Fig. 1. Bootstrap-based cluster stability analyses indicated heterogeneous stability across the seven-cluster solution. Mean Jaccard coefficients for clusters 1–7 were 0.75, 0.61, 0.52, 0.87, 0.46, 0.65, and 0.54, respectively, suggesting good stability for Cluster 4, moderate stability for Clusters 1, 2, 6, and 7, and comparatively low stability for Clusters 3 and 5.

**Table 1:** Sample characteristics by child maltreatment profiles derived from the dimensions of the Childhood Trauma Questionnaire. PDD = persistent depressive disorder; PD = personality disorder; BDI-II = Beck Depression Inventory, 2nd version; BSL-23 = Borderline Symptom List.

Variable	Type 1 (*n* = 69)	Type 2 (*n* = 49)	Type 3 (*n* = 30)	Type 4 (*n* = 32)	Type 5 (*n* = 25)	Type 6 (*n* = 27)	Type 7 (*n* = 8)	Total (*n* = 240)
Male gender, %, n	36.8, 25	46.9, 23	23.3, 7	21.9, 7	16.7, 4	26.9, 7	12.5, 1	31.2, 74
Female gender, %, n	61.8, 42	53.1, 26	73.3, 22	75.0, 24	83.3, 20	73.1, 19	87.5, 7	67.5, 160
Diverse gender, %, n	1.5, 1	0.0, 0	3.3, 1	3.1, 1	0.0, 0	0.0, 0	0.0, 0	1.3, 3
Age, M (SD), n	32.1 (12.1), 62	36.1 (13.1), 48	29.2 (10.8), 30	33.8 (12.9), 32	32.5 (11.0), 24	36.1 (15.9), 27	34.7 (10.7), 8	33.4 (12.7), 231
PDD diagnosis, %, n	59.4, 41	73.5, 36	50.0, 15	56.2, 18	36.0, 9	59.3, 16	37.5, 3	57.5, 138
Married, %, n	14.9, 10	17.0, 8	6.9, 2	25.0, 6	0.0, 0	20.0, 4	14.3, 1	14.3, 31
Employed, %, n	34.4, 22	46.8, 22	34.5, 10	39.1, 9	36.4, 8	52.6, 10	0.0, 0	38.4, 81
Outpatient treatments, M (SD), n	2.3 (1.4), 50	1.8 (1.3), 36	1.8 (0.8), 23	2.0 (1.2), 21	1.7 (1.2), 15	1.8 (0.9), 15	2.2 (0.8), 5	2.0 (1.2), 165
Inpatient treatments, M (SD), n	2.8 (1.9), 63	2.6 (2.3), 42	2.7 (1.9), 26	3.3 (2.8), 21	3.4 (2.7), 22	3.5 (2.0), 17	4.8 (3.3), 6	3.0 (2.3), 197
BDI-II, M (SD), n	30.2 (10.5), 69	28.8 (9.3), 49	36.6 (8.7), 30	35.6 (8.5), 32	33.8 (7.1), 25	34.5 (9.6), 27	41.8 (8.9), 8	32.7 (9.8), 240
BSL-23, M (SD), n	1.5 (0.7), 69	1.3 (0.6), 49	1.9 (0.7), 30	2.0 (0.8), 32	1.7 (0.6), 25	1.5 (0.8), 27	2.7 (0.8), 8	1.6 (0.7), 240

### Lifetime and current cross-sectional suicidal ideations and behaviors according to child maltreatment profiles

CM clusters were significantly associated with the lifetime occurrence of active SI with plan and intent, SB without actual suicide attempt (preparations of suicide attempts, aborted or interrupted attempts), SB with potentially lethal suicide attempts, and lifetime severity ranks of SI and SB (Table 2). Pairwise comparisons (see Supplementary Table 2 including odds ratios and confidence intervals) revealed significantly more frequent lifetime occurrences of SB, including potentially lethal attempts (OR: 8.8, *p*_*FDR*_=0.002, 95% CI [2.9, 25.9] vs. Cluster 2) and higher lifetime SB severity ranks in type 6 compared to types 1, 2, and 5. Type 6 showed higher lifetime occurrence of active SI with plan and intent (OR: 5.0, *p*_*FDR*_ = 0.038, 95% CI [1.8, 13.7]) and lifetime SI severity rank (OR: 5.4, p_FDR_ = 0.010, 95% CI [2.0, 13.9]) than type 2. Similar, yet slightly smaller effect sizes were found for comparisons involving type 7, however, contrasts were shy of statistical significance due to small cell sizes. We did not find significant differences of current SIB at cross-sectional pre or post time points according to CM clusters. Due to low observed base rates, particularly in acute SB (only 15% of participants) we carried out post-hoc power analysis for the acute SIB models at baseline. Assuming a significancy level of alpha = 0.05, our sample size of *n* = 240 would have been able to detect a medium effect of Cohen’s w = 0.24 with 1-β = 0.80 for a cumulative link regression model comparing 7-levels of CM clusters. For the observed effect sizes our models reached a power of 0.48 for acute SI (w=0.17) and 0.12 for acute SB (w=0.08).

**Table 2:** Suicidal ideations and behaviors by profiles derived from the dimensions of the Childhood Trauma Questionnaire. SIB = suicidal ideation and behavior; SI = suicidal ideation; SB = suicidal behavior; actual suicide attempts = potentially lethal; n.a. = not available due to small cell size; Analyses for acute SIB at baseline or post time points were controlled for lifetime SIB; For this purpose, we added the CTQ cluster variable to a base model including lifetime SIB; P-values for acute SIB models reflect the statistical significance of the additional contribution of the CTQ cluster beyond the base model.

Type	Time	Variable	Type 1 (*n* = 69)	Type 2 (*n* = 49)	Type 3 (*n* = 30)	Type 4 (*n* = 32)	Type 5 (*n* = 25)	Type 6 (*n* = 27)	Type 7 (*n* = 8)	Total (*n* = 240)	χ2(6)	*P*
SI	Life-time	passive, n (%)	60 (87.0)	36 (73.5)	25 (83.3)	28 (87.5)	22 (88.0)	25 (92.6)	7 (87.5)	203 (84.6)	6.43	0.377
		unspecific active, n (%)	53 (76.8)	28 (57.1)	21 (70.0)	22 (71.0)	18 (72.0)	23 (85.2)	6 (75.0)	171 (71.5)	8.49	0.204
		active with plan or intent, n (%)	47 (68.1)	22 (44.9)	20 (66.7)	20 (62.5)	15 (60.0)	21 (77.8)	6 (75.0)	151 (62.9)	10.92	0.091
		active with plan and intent, n (%)	29 (42.6)	14 (28.6)	13 (43.3)	12 (37.5)	7 (28.0)	18 (66.7)	5 (62.5)	98 (41.0)	14.12	0.028*
		ordinal [none], n (%)	9 (13.0)	13 (26.5)	5 (16.7)	3 (9.4)	3 (12.0)	2 (7.4)	1 (12.5)	36 (15.0)	14.67	0.023*
		ordinal [passive], n (%)	6 (8.7)	8 (16.3)	4 (13.3)	7 (21.9)	4 (16.0)	2 (7.4)	1 (12.5)	32 (13.3)		
		ordinal [unspecific active], n (%)	6 (8.7)	6 (12.2)	1 (3.3)	2 (6.2)	2 (8.0)	2 (7.4)	0 (0.0)	19 (7.9)		
		ordinal [active with plan or intent], n (%)	19 (27.5)	8 (16.3)	7 (23.3)	8 (25.0)	9 (36.0)	3 (11.1)	1 (12.5)	55 (22.9)		
		ordinal [active with plan and intent], n (%)	29 (42.0)	14 (28.6)	13 (43.3)	12 (37.5)	7 (28.0)	18 (66.7)	5 (62.5)	98 (40.8)		
	Base-line	ordinal [none], n (%)	28 (40.6)	31 (63.3)	15 (50.0)	13 (40.6)	11 (44.0)	12 (44.4)	2 (25.0)	112 (46.7)	4.07	0.667
		ordinal [passive], n (%)	23 (33.3)	11 (22.4)	8 (26.7)	13 (40.6)	9 (36.0)	10 (37.0)	5 (62.5)	79 (32.9)		
		ordinal [unspecific active], n (%)	4 (5.8)	4 (8.2)	2 (6.7)	0 (0.0)	1 (4.0)	2 (7.4)	1 (12.5)	14 (5.8)		
		ordinal [active with plan or intent], n (%)	14 (20.3)	2 (4.1)	5 (16.7)	5 (15.6)	4 (16.0)	2 (7.4)	0 (0.0)	32 (13.3)		
		ordinal [active with plan and intent], n (%)	0 (0.0)	1 (2.0)	0 (0.0)	1 (3.1)	0 (0.0)	1 (3.7)	0 (0.0)	3 (1.2)		
	Post	ordinal [none], n (%)	32 (52.5)	30 (73.2)	13 (59.1)	14 (56.0)	15 (78.9)	14 (70.0)	4 (57.1)	122 (62.6)	7.03	0.318
		ordinal [passive], n (%)	13 (21.3)	8 (19.5)	6 (27.3)	7 (28.0)	1 (5.3)	4 (20.0)	2 (28.6)	41 (21.0)		
		ordinal [unspecific active], n (%)	8 (13.1)	2 (4.9)	3 (13.6)	0 (0.0)	0 (0.0)	1 (5.0)	0 (0.0)	14 (7.2)		
		ordinal [active with plan or intent], n (%)	8 (13.1)	1 (2.4)	0 (0.0)	4 (16.0)	3 (15.8)	1 (5.0)	1 (14.3)	18 (9.2)		
SB	Life-time	suicidal behavior without attempt, n (%)	34 (49.3)	20 (40.8)	19 (63.3)	18 (56.2)	9 (36.0)	20 (74.1)	6 (75.0)	126 (52.5)	14.34	0.026*
		suicidal behavior with attempt, n (%)	23 (33.3)	12 (24.5)	15 (50.0)	14 (43.8)	8 (32.0)	20 (74.1)	4 (50.0)	96 (40.0)	21.87	0.001**
		ordinal [none], n (%)	30 (43.5)	26 (53.1)	9 (30.0)	11 (34.4)	13 (52.0)	5 (18.5)	1 (12.5)	95 (39.6)	20.34	0.002**
		ordinal [behavior without attempt], n (%)	16 (23.2)	11 (22.4)	6 (20.0)	7 (21.9)	4 (16.0)	2 (7.4)	3 (37.5)	49 (20.4)		
		ordinal [behavior with attempt], n (%)	23 (33.3)	12 (24.5)	15 (50.0)	14 (43.8)	8 (32.0)	20 (74.1)	4 (50.0)	96 (40.0)		
	Base-line	ordinal [none], n (%)	60 (87.0)	41 (83.7)	24 (80.0)	28 (87.5)	22 (88.0)	22 (81.5)	7 (87.5)	204 (85.0)	2.76	0.838
		ordinal [behavior without attempt], n (%)	9 (13.0)	5 (10.2)	5 (16.7)	2 (6.2)	2 (8.0)	3 (11.1)	1 (12.5)	27 (11.2)		
		ordinal [behavior with attempt], n (%)	0 (0.0)	3 (6.1)	1 (3.3)	2 (6.2)	1 (4.0)	2 (7.4)	0 (0.0)	9 (3.8)		
	Post	ordinal [none], n (%)	59 (96.7)	41 (100.0)	22 (100.0)	24 (96.0)	17 (100.0)	19 (95.0)	6 (85.7)	188 (97.4)	n.a.	n.a.
		ordinal [behavior without attempt], n (%)	2 (3.3)	0 (0.0)	0 (0.0)	1 (4.0)	0 (0.0)	1 (5.0)	0 (0.0)	4 (2.1)		
		ordinal [behavior with attempt], n (%)	0 (0.0)	0 (0.0)	0 (0.0)	0 (0.0)	0 (0.0)	0 (0.0)	1 (14.3)	1 (0.5)		

### Suicidal ideation and behavior changes during inpatient psychotherapy according to child maltreatment profiles

**Fig. 2:**
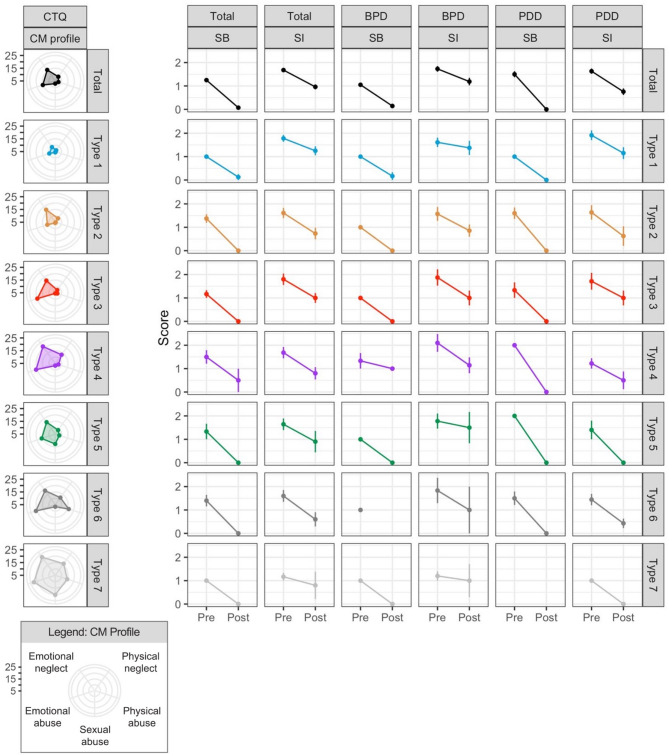
Changes of suicidal ideation and behavior scores from pre to post time points according to child maltreatment clusters. Childhood Trauma Questionnaire (CTQ) scores served as the basis for defining clusters of child maltreatment, which were identified through agglomerative hierarchical clustering. CTQ patterns, labeled types 1–7, are distinguished by varying combinations of maltreatment dimensions; SB = suicidal behaviors; SI = suicidal ideations; BPD = borderline personality disorder; PDD = persistent depressive disorder.

To investigate the change of SIB to inpatient psychotherapy programs, we analyzed only participants who reported current SIB at baseline (Fig. 2, Supplementary Table 3). One hundred and twenty-eight participants with current SI (BPD: 63, PDD: 65) and 36 participants with current SB (BPD: 20, PDD: 16) were available for analysis. A significant time effect for SI was found after CBASP (F_(1, 48.11)_ = 17.86, *p* < .001, d_paired_ = 0.69, 95% CI [0.39, 0.99]) and DBT (F_(1, 49.05)_ = 7.75, *p* = .008, d_paired_ = 0.34, 95% CI [0.05, 0.63]), reflective of a reduction in SI across clusters. There was no significant time x CM cluster interaction (CBASP: F_(6, 50.13)_ = 0.33, *p* = .917, *ω*² = 0.00; DBT: F_(6, 48.11)_ = 0.41, *p* = .870, *ω*² = 0.00). Similarly, significant time effects were found for SB (CBASP: F_(1, 8.14)_ = 83.46, *p* < .001; DBT: F_(1, 7.39)_ = 58.39, *p* < .001), but no significant time x CM cluster interaction (CBASP: F_(5, 8.28)_ = 0.71, *p* = .635; DBT: F_(5, 7.04)_ = 0.93, *p* = .516) with all but 2 (7%) participants showing no more SB after treatment. However, due to only 2 non-zero SB values at post treatment, the parameters within clusters could not be uniquely determined.

### Disorder-specific symptom changes during inpatient psychotherapy according to child maltreatment clusters

**Fig. 3:**
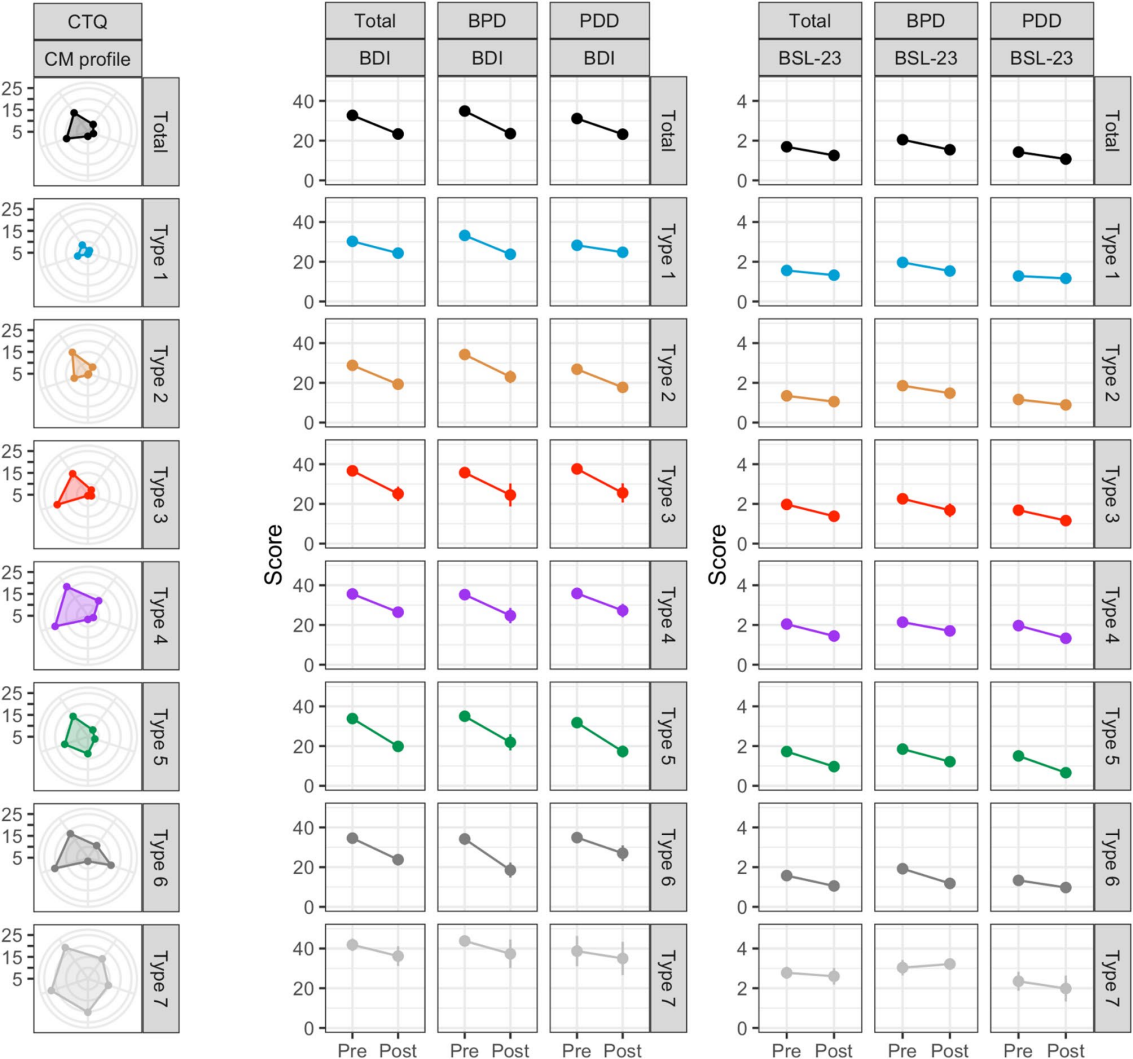
Changes of disorder-specific symptom scores from pre to post time points according to child maltreatment clusters. CTQ scores served as the basis for defining clusters of child maltreatment, which were identified through agglomerative hierarchical clustering. CM profiles, labeled types 1–7, are distinguished by varying combinations of maltreatment dimensions; BDI = Beck Depression Inventory 2nd version; BSL-23 = Borderline Symptom List 23, BPD = borderline personality disorder; PDD = persistent depressive disorder.

Significant time effects on the BDI-II and BSL-23 were found after both treatments (*p* < .05). While no between-cluster differences were found after DBT, we found significant time x CM cluster interactions on both measures after CBASP (BDI-II: F_(6, 134.65)_ = 2.82, *p* = .013, *ω*² = 0.07; BSL-23: F_(6, 236)_ = 2.32, *p* = .034, *ω*² = 0.03) (Fig. 3, Supplementary Table 4). After CBASP, patients in cluster type 1 (no CM) and type 7 (emotional neglect and emotional as well as sexual abuse) showed only little improvement in depressive symptoms compared with the other clusters. Pairwise comparisons revealed significant differences between type 1 and types 2–6 (Supplementary Table 5), while comparisons with type 7 were not significant due to the limited sample size in that cluster. Improvements on borderline symptoms after CBASP were strongest in type 4 (predominant emotional neglect and emotional abuse) and type 5 (predominant emotional neglect, emotional and sexual abuse) with both clusters being superior to type 1 and type 5 also being superior to type 2.

### Comparison of predictability between child maltreatment clusters and total score

Overall similar but low predictive accuracy for CM clusters and CTQ total scores was observed for the lifetime and acute SIB measures. Detailed model characteristics are reported in the Supplement (Supplementary Table 6).

## Discussion

This observational cohort study investigated differences in lifetime and acute SIB as well as symptom change during disorder-specific inpatient psychotherapy programs by profiles of CM in a high-risk cross-diagnostic sample. The main finding was that CM profiles can effectively detect risk for lifetime SIB, i.e., SI with plan and intent, and SB including potentially lethal suicide attempts. No differences in acute SIB were found between CM profiles. Following inpatient psychotherapy programs, SIB were significantly reduced with no differential effects across CM profiles. In contrast, CM profiles were associated with amelioration of disorder-specific symptoms after CBASP, but not after DBT.

The seven CM clusters and their characteristics identified in the present sample, were previously found in independent samples^21,22^ and in a recent cross-culture replication by an independent group^23^. However, classical cluster validity indices indicated only modest support for a strongly separated cluster structure. This is not unexpected, as CTQ responses likely reflect graded and overlapping patterns of adversity rather than sharply bounded latent subgroups. As such, CM clusters need to still be considered exploratory and not generalizable to date.

CM profiles were associated with a differential risk for active components of lifetime SIB. Cluster 6 showed a significant association with lifetime SI with plan and intent, and SB including potentially lethal suicide attempts. These results are consistent with previous research suggesting that cumulative and severe forms of CM can contribute to an elevated SIB risk^4–6,9,12,18,19,27^. In contrast, profiles characterized by minimal or no CM exposure were generally associated with lower lifetime SIB risk, which reinforces the notion that the intensity and complexity of CM experiences critically influence mental health outcomes^12,18^. Our results regarding the prominent role of cluster 6 suggest that particularly experiences of physical abuse may contribute to the risk of active SIB. While there are few studies who used a nuanced assessment of SIB, thus allowing to differentiate between risk factors for passive versus active components of SIB, this finding is in line with one previous study that found an elevated risk for active components of SIB in people with experiences of childhood physical abuse^6^. Theoretically, our findings align with interpersonal theories of suicide suggesting that previous experiences of physical violence reduce the threshold to inflict violence upon oneself^28^.

Overall, our findings suggest that lifetime manifestations of suicidality, rather than cross-sectional acute SIB, may be more closely associated with CM profiles. However, given the limited statistical power of this sample regarding acute SIB (see post hoc power analysis), the null findings for acute SIB should not be interpreted as evidence of absence but rather exploratory and hypotheses generating. Together with floor-effects, which are inherent to the rare nature of acute and severe forms of SIB, as well as restricted variability inherent to cross-sectional measurements, this hampers conclusions that can be drawn from these negative findings and highlights the importance of larger high-risk samples to increase the likelihood of observing acute and severe forms of SIB as well as incorporating dynamic risk monitoring approaches, such as experience sampling methods, that can capture temporal fluctuations in risk factors, complementing CM profiling.

Following inpatient psychotherapy programs, SIB scores were reduced compared to baseline. The overall decline of SIB following inpatient psychotherapy programs is broadly consistent with a recent meta-analysis indicating that indirect psychotherapy, such as CBASP or DBT, is similarly effective in reducing SIB as psychotherapy targeted directly at SIB^3^. However, conclusions regarding the therapeutic effects of the two inpatient psychotherapy programs are hampered by the lack of comparator conditions. In addition, analyses restricted to participants with current SIB at baseline are susceptible to regression to the mean, which may contribute to pre–post decreases independent of specific treatment effects, thereby limiting any inference regarding treatment effects.

Clinical symptoms showed a differential change during CBASP depending on the CM profile. Patients with moderate-to-severe CM histories (profiles type 2–6) demonstrated greater improvements of depressive symptoms than those without CM (cluster type 1), while patients with combined emotional and sexual abuse (cluster type 7) showed more limited benefit. Notably, such differential changes were not observed after DBT. These findings are consistent with previous work showing that individuals with depression and elevated CM benefited more from CBASP in comparison to supportive psychotherapy^22^. The stronger improvements after CBASP in clusters with higher CM burden (except cluster type 7) would match CBASP’s focus on early interpersonal trauma, which is conceptually embedded in its treatment model - unlike DBT. Importantly, lower improvements in cluster type 1 cannot be attributed to floor effects, as baseline symptom levels were clinically relevant. However, interpretations of differential symptom change between CBASP and DBT should be made with caution, as treatment allocation was confounded with diagnosis (CBASP for PDD; DBT for BPD), and no formal interaction model including treatment × CM profile × time across both treatments was tested. Additionally, differential symptom changes across CM profiles may partly reflect differences in baseline severity and potential regression to the mean, particularly in groups with higher initial symptom burden. The limited change in cluster type 7 should be interpreted cautiously given the small sample size and resulting imprecision of estimates. In sum, this study provides preliminary evidence, that CM profiling may inform depression treatment personalization with respect to CBASP but the relative suitability of specific treatments for particular CM profiles remains tentative and warrants replication in future studies.

Finally, an important conceptual question concerns whether CM is best represented as a continuum of increasing severity or as discrete profiles. In our data, several clusters appear to follow a gradient of increasing CM burden; however, they also differ in the composition of CM subtypes, particularly with respect to the presence of physical or sexual abuse. This configurational perspective may provide clinically relevant information beyond unidimensional severity scores^23^. However, in this study, CM cluster-based models did not demonstrate superior predictive validity for SIB outcomes compared with a continuous CM score. Overall model performance was low for both CM sum scores and clusters, likely reflecting the limitation of relying on a single predictor to model multifactorial SIB outcomes or limited predictive information in CM for SIB outcomes. Although these findings may tentatively support the clinical utility of continuous CM scores, they should be interpreted with caution, as robust evaluation requires benchmarking in larger, more representative samples.

While our study shows methodological strength in an innovative approach to assessing complex CM profiles, a differential measure of SIB (C-SSRS), and a longitudinal observational design of a high-risk sample in clinical routine, it has methodological limitations that have partly been discussed previously and are summarized in the following. First, the reliance on CTQ information to assess CM may have neglected other relevant CM experiences (e.g., witnessing partner violence or bullying). Second, the CM profiling approach was developed in this specific sample and may not generalize to other populations, limiting its broader applicability. Third, the lack of control conditions regarding the numerous confounders of inpatient psychotherapy programs (e.g., effects of therapeutic milieu, structured daily routines, or social support) hampers conclusions regarding treatment effects, which are further limited by potential regression to the mean, which may occur specifically in groups with high initial symptom burden. Fourth, the moderate sample size of patients reporting current SIB before treatment resulted in small samples for distinct profiles, particularly type 7, when predicting changes in SIB after treatment, limiting its further interpretation. Similarly, current SIB at baseline and post-treatment time points showed floor effects at a limited sample size, and the lack of a significant association between current cross-sectional SIB and CM profiles should not be interpreted as negative evidence. Fifth, the use of convenience sampling, the restriction to individuals with BPD and PDD, excluding acute mania, psychotic disorders, or severe substance use disorders, as well as sample-specific comorbidity limit the generalizability of our findings to broader clinical populations, particularly those with other psychiatric conditions in which suicidality is prevalent.

In conclusion, this study provides preliminary evidence that profiles of early life adversity based on CM clustering may offer an additional approach to assessing the lifetime risk of active SI with plan and intent as well as potentially lethal suicide attempts in high-risk populations with PDD and BPD. While CM patterns were not associated with differential SIB changes after disorder-specific inpatient psychotherapy programs, SIB decreased significantly across all CM clusters. Following CBASP, individuals with CM histories showed greater reductions in depressive symptoms compared to those without CM, which may suggest that CM profiling could aid in personalizing depression treatment. However, interpretations remain tentative given the exploratory nature of the study. These exploratory findings warrant replication to potentially support the integration of CTQ based CM profiling into clinical routines as an adjunct to inform lifetime SIB risk assessment as well as personalized depression treatment. Future research should focus on developing a generalizable and robust approach of CM profiling in larger more diverse cohorts, systematically comparing cluster-based and continuous representations of CM in larger samples, and exploring its potential to guide clinical decisions and routines in primary and specialized care.

Methods.

### Design and setting

This was a single-site observational cohort study conducted within two inpatient psychotherapy programs, i.e., Dialectical Behavioral Therapy (DBT) for BPD and CBASP for PDD, at the Department for Psychiatry and Psychotherapy of the LMU University Hospital in Munich, Germany. The study adhered to the principles outlined in the Declaration of Helsinki and received approval from the Research Ethics Board of the LMU, Faculty of Medicine, Munich (approval number #713–15; study registration DRKS00019821).

### Participants

A sample of 288 adults were recruited between June 2018 and October 2024 utilizing convenience sampling for its efficiency. Patients were eligible if they met DSM-5 criteria for BPD or PDD and provided written informed consent. Diagnoses were assigned following DSM-5 criteria through structured clinical interviews (SCID-I/-II, SCID-5-CV/-PD, respectively). Exclusion criteria included mania, psychosis, substance use disorders as a primary diagnosis, pregnancy, or acute somatic conditions. Race and ethnicity were not routinely collected. For this study, only participants with complete datasets of the Columbia-Suicide Severity Rating Scale (C-SSRS)^29^ and the CTQ were included, resulting in a maximum available dataset of 240 participants. Because no a priori power analysis was performed, we computed the minimum required effect sizes our observed sample size was able to detect with adequate power. Assuming a power of 80%, a sample size of 240 participants was sufficient to detect at least a medium effect size of f = 0.24 for the cross-sectional test of the seven-level CM profile factor and f = 0.12 for the time x CM profile interaction. A CONSORT chart of participants is available in Supplementary Fig. 1.

### Procedure

Each group underwent a 10-week inpatient psychotherapy program. The PDD group received CBASP modified and manualized for a multidisciplinary inpatient treatment^30^. It included the following CBASP modules: individual therapy (2 sessions of 50 min per week) and group therapy (2 sessions of 100 min per week). BPD patients received DBT with a comparable therapeutic dose following established protocols of DBT inpatient treatment^31,32^. In both groups, psychopharmacological medication remained stable or was optimized according to current German guidelines. All measures were taken before and after the 10-week inpatient treatment programs.

### Measures

To assess SIB as our primary outcome, we used the German version of the C-SSRS. Main outcomes included lifetime scores for suicidal ideation (SI) and suicidal behaviors (SB), as well as current SI and SB assessed for the week before and the week after treatment. For each time point, the most severe level of SI and SB was recorded with binary variables (yes, no) across five severity levels: from passive SI to active SI with plan and intent and from preparatory SB to completed suicide. Categorical SI and SB variables were created by aggregating the binary variables into an ordinal SI and SB severity score, with higher scores reflecting greater severity. As both SI and SB were assessed on ordinal scales, internal consistency analysis was not performed in line with the recommendations by Posner et al. 2011.

As secondary outcomes, the severity of depressive symptoms was measured using the German version of the Beck Depression Inventory 2nd version (BDI-II)^33,34^(α = 0.96)^31^ and the severity of borderline symptoms was measured with the short version of the Borderline Symptom List (BSL-23; α = 0.93)^35^. Higher scores indicate more severe symptoms.

CM recall was assessed with the German version of the CTQ (CTQ-SR), comprising 28 items rated on 5-point Likert Scales^36,37^. Sum scores were computed for sexual abuse (α = 0.89), physical abuse (α = 0.80), emotional abuse (α = 0.87), physical neglect (α = 0.55), and emotional neglect (α = 0.83) experienced before the age of eighteen^38^. Higher scores indicate more frequent CM.

### Analyses

Statistical analyses were performed in R, version 4.4.1. Results were considered statistically significant at $\:\alpha\:$ = 0.05. For the agglomerative hierarchical clustering algorithm (stats::hclust function), the five CTQ subscales were utilized to categorize individuals into CM clusters similar to Goerigk et al.^21,22^ using the ward.D2 method on the Euclidean distance and an adaptive branch pruning procedure to determine the number of clusters to retain. Cluster stability of the selected seven-cluster solution was evaluated using bootstrap resampling (B = 1000) and stability was quantified using clusterwise mean Jaccard coefficients obtained via the clusterboot procedure^39^. Binary outcomes (lifetime occurrences of SIB) were modeled using logistic regression and ordinal outcomes (lifetime SIB severity rank) were modeled using cumulative link regression models (ordinal::clm function). To evaluate treatment effects, repeated measures of SI, SB, depressive (BDI-II) and borderline (BSL-23) symptoms were modeled separately for both treatment groups using linear mixed regression models including time (pre vs. post), CM cluster (types 1–7) and their interaction as well as the baseline symptom severity (BDI-II or BSL-23 scores) as covariates. Significance of model factors was determined using Type-III analyses of variance. Effect sizes for model factors were reported as partial ω²; negative partial ω² estimates were truncated to zero^40^. Finally, we compared the predictive utility of CM clusters with a simpler CM sum score for SIB outcomes; the used methods are described in detail in the supplement. Analyses were performed on complete cases without imputation. P-values of multiple comparisons were adjusted using false-discovery-rate (FDR) correction with the Benjamini-Hochberg method^41^.

## Supplementary Information

Below is the link to the electronic supplementary material.

Supplementary Material 1

## Data Availability

Individual-level participant data are not publicly available because of the sensitive nature of the clinical and trauma-related information and restrictions imposed by the ethics committee and national data protection regulations. Deidentified aggregated data underlying the main analyses and a detailed description of the analytic code can be made available to qualified researchers upon reasonable request to the corresponding author, subject to approval by the local ethics committee and completion of a data use agreement.
