# Supplementary material for: Lifetime and acute risk of suicidality by profiles of early life adversity: an observational cohort study in a high-risk population
Source: Sci Rep. 2026 Jul 1;16:20155. doi: 10.1038/s41598-026-60185-7 (PMC13324715; doi:10.1038/s41598-026-60185-7)
Supplement: Supplementary file 1 — Supplementary Material 1 [file 41598_2026_60185_MOESM1_ESM.pdf]

Supplement

Supplementary Table 1. Sample comorbidity

**Comorbidity, n (%)**	**BP (n=102)**	**CD (n=138)**	**Total (n=240)**
Affective disorder[1]	69 (67.6)	112 (81.1)	181 (75.4)
Anxiety disorder	54 (52.9)	67 (48.5)	121 (50.4)
Obsessive-compulsive disorder	10 (9.8)	11 (7.9)	21 (8.7)
Post-traumatic stress disorder	43 (42.1)	22 (15.9)	65 (27.0)
Eating disorder	25 (24.5)	10 (7.2)	35 (14.5)
Personality disorder	43 (42.1)	55 (39.8)	98 (40.8)

Note: Comorbidity categories were based on current diagnoses assessed with SCID-I or SCID-5-CV where available. Affective disorders comprised major depressive episode, hypomania, bipolar I disorder, bipolar II disorder, and unspecified affective disorders. Anxiety disorders comprised panic disorder, agoraphobia, social anxiety disorder, specific phobia, and generalized anxiety disorder, and eating disorders comprised anorexia nervosa, bulimia nervosa, and binge-eating disorder.

Supplementary Table 2. Pairwise comparisons of SIB variables between CM clusters derived from the dimensions of the Childhood Trauma Questionnaire

	**Suicidal ideation**						**Suicidal behavior**								
	**Active with plan and intent**			**Lifetime severity rank**			**Without attempt**			**With attempt**			**Lifetime severity rank**		
**Contrast**	**OR**	**P**	**P_{FDR}**	**OR**	**P**	**P_{FDR}**	**OR**	**P**	**P_{FDR}**	**OR**	**P**	**P_{FDR}**	**OR**	**P**	**P_{FDR}**
Type 2 - Type 1	0.54 (0.25 - 1.18)	0.121	0.318	0.44 (0.22 - 0.85)	0.015*	0.151	0.71 (0.34 - 1.49)	0.364	0.506	0.65 (0.29 - 1.47)	0.302	0.482	0.67 (0.34 - 1.34)	0.263	0.394
Type 3 - Type 1	1.03 (0.43 - 2.45)	0.95	0.959	0.91 (0.42 - 2.01)	0.824	0.824	1.78 (0.74 - 4.29)	0.2	0.350	2 (0.84 - 4.79)	0.12	0.315	1.9 (0.85 - 4.26)	0.12	0.234
Type 3 - Type 2	1.91 (0.74 - 4.95)	0.182	0.403	2.1 (0.91 - 4.86)	0.084	0.220	2.5 (0.98 - 6.39)	0.055	0.229	3.08 (1.17 - 8.11)	0.023*	0.095	2.81 (1.19 - 6.68)	0.019*	0.099
Type 4 - Type 1	0.81 (0.34 - 1.91)	0.626	0.747	0.82 (0.39 - 1.73)	0.6	0.742	1.32 (0.57 - 3.07)	0.515	0.630	1.56 (0.66 - 3.67)	0.314	0.482	1.51 (0.69 - 3.28)	0.304	0.425
Type 4 - Type 2	1.5 (0.58 - 3.87)	0.401	0.562	1.88 (0.85 - 4.17)	0.122	0.284	1.86 (0.76 - 4.59)	0.176	0.350	2.4 (0.92 - 6.23)	0.073	0.218	2.23 (0.97 - 5.16)	0.06	0.181
Type 4 - Type 3	0.78 (0.28 - 2.17)	0.64	0.747	0.9 (0.36 - 2.21)	0.811	0.824	0.74 (0.27 - 2.06)	0.57	0.630	0.78 (0.29 - 2.11)	0.622	0.726	0.79 (0.31 - 2.02)	0.627	0.693
Type 5 - Type 1	0.52 (0.19 - 1.42)	0.202	0.403	0.7 (0.32 - 1.54)	0.371	0.590	0.58 (0.23 - 1.49)	0.256	0.414	0.94 (0.35 - 2.5)	0.903	0.948	0.79 (0.33 - 1.9)	0.601	0.693
Type 5 - Type 2	0.97 (0.33 - 2.84)	0.959	0.959	1.6 (0.69 - 3.7)	0.275	0.524	0.82 (0.3 - 2.21)	0.688	0.723	1.45 (0.5 - 4.2)	0.493	0.608	1.17 (0.47 - 2.96)	0.734	0.734
Type 5 - Type 3	0.51 (0.16 - 1.58)	0.242	0.424	0.76 (0.3 - 1.96)	0.572	0.742	0.33 (0.11 - 0.98)	0.046*	0.229	0.47 (0.16 - 1.42)	0.181	0.38	0.42 (0.15 - 1.15)	0.092	0.214
Type 5 - Type 4	0.65 (0.21 - 2)	0.452	0.593	0.85 (0.34 - 2.11)	0.727	0.824	0.44 (0.15 - 1.28)	0.132	0.345	0.61 (0.2 - 1.8)	0.367	0.482	0.53 (0.19 - 1.42)	0.206	0.332
Type 6 - Type 1	2.69 (1.06 - 6.84)	0.038*	0.198	2.35 (0.96 - 5.79)	0.062	0.220	2.94 (1.1 - 7.85)	0.031*	0.219	**5.71 (2.11 - 15.47)**	**0.001****	**0.006****	**5.09 (1.93 - 13.4)**	**0.001****	**0.01***

Type 6 - Type 2	5 (1.82 - 13.76)	0.002**	0.038*	5.4 (2.09 - 13.95)	<.001***	0.010*	4.14 (1.48 - 11.63)	0.007**	0.076	8.81 (2.99 - 25.92)	<.001***	0.002**	7.54 (2.73 - 20.86)	<.001***	0.002**
Type 6 - Type 3	2.62 (0.89 - 7.68)	0.08	0.267	2.57 (0.92 - 7.24)	0.073	0.220	1.65 (0.53 - 5.16)	0.386	0.506	2.86 (0.93 - 8.75)	0.066	0.218	2.68 (0.9 - 8.01)	0.078	0.204
Type 6 - Type 4	3.33 (1.14 - 9.75)	0.028*	0.196	2.87 (1.05 - 7.84)	0.039*	0.206	2.22 (0.73 - 6.73)	0.158	0.350	3.67 (1.21 - 11.13)	0.021*	0.095	3.38 (1.15 - 9.92)	0.027*	0.112
Type 6 - Type 5	5.14 (1.57 - 16.81)	0.007**	0.071	3.38 (1.2 - 9.55)	0.022*	0.151	5.08 (1.55 - 16.64)	0.007**	0.076	6.07 (1.82 - 20.22)	0.003**	0.023*	6.42 (2.04 - 20.28)	0.002**	0.011*
Type 7 - Type 1	2.24 (0.5 - 10.14)	0.295	0.476	1.82 (0.41 - 8.05)	0.432	0.605	3.09 (0.58 - 16.38)	0.185	0.350	2 (0.46 - 8.73)	0.357	0.482	2.51 (0.67 - 9.49)	0.174	0.304
Type 7 - Type 2	4.17 (0.88 - 19.82)	0.073	0.267	4.17 (0.91 - 19.04)	0.066	0.220	4.35 (0.8 - 23.78)	0.09	0.269	3.08 (0.67 - 14.26)	0.15	0.349	3.73 (0.95 - 14.57)	0.059	0.181
Type 7 - Type 3	2.18 (0.44 - 10.83)	0.341	0.511	1.99 (0.41 - 9.59)	0.393	0.590	1.74 (0.3 - 10.14)	0.54	0.630	1 (0.21 - 4.76)	1	1	1.32 (0.32 - 5.5)	0.7	0.734
Type 7 - Type 4	2.78 (0.56 - 13.76)	0.211	0.403	2.22 (0.47 - 10.5)	0.315	0.552	2.33 (0.41 - 13.37)	0.342	0.506	1.29 (0.27 - 6.07)	0.751	0.83	1.67 (0.41 - 6.84)	0.476	0.588
Type 7 - Type 5	4.29 (0.8 - 22.92)	0.089	0.267	2.61 (0.54 - 12.63)	0.234	0.491	5.33 (0.88 - 32.16)	0.068	0.237	2.12 (0.42 - 10.75)	0.362	0.482	3.17 (0.73 - 13.74)	0.122	0.234
Type 7 - Type 6	0.83 (0.16 - 4.3)	0.827	0.915	0.77 (0.15 - 3.94)	0.755	0.824	1.05 (0.17 - 6.46)	0.958	0.958	0.35 (0.07 - 1.79)	0.207	0.396	0.49 (0.11 - 2.26)	0.363	0.476

Note: SIB = suicidal ideation and behavior; OR odds ratio with 95% confidence interval, numbers > 1 indicate higher odds of the first named category for lifetime occurrence for binary variables and higher odds to be assigned to a higher severity rank for ordinal variables; FDR false-discovery-rate corrected * $p < .05$, ** $p < .01$, *** $p < .001$.

Supplementary Table 3. SI and SB before and after treatment

	Suicidal behavior						Suicidal ideation					
	BPD			PDD			BPD			PDD		
**Cluster**	**Pre**	**Post**	**ES**	**Pre**	**Post**	**ES**	**Pre**	**Post**	**ES**	**Pre**	**Post**	**ES**
Type 1	1 (0)	0.17 (0.41)	2.04 (0.55 to 3.48)	1 (0)	0 (0)		1.61 (0.85)	1.38 (1.2)	0.14 (-0.35 to 0.63)	1.91 (1)	1.15 (1.14)	0.46 (-0.01 to 0.91)
Type 2	1 (0)	0 (0)		1.6 (0.55)	0 (0)	2.92 (0.78 to 5.04)	1.57 (0.79)	0.86 (0.69)	0.75 (-0.12 to 1.58)	1.64 (1.03)	0.62 (1.19)	0.94 (0.07 to 1.76)
Type 3	1 (0)	0 (0)		1.33 (0.58)	0 (0)	2.12 (-0.65 to 5.01)	1.88 (0.99)	1 (0.71)	0.61 (-0.38 to 1.55)	1.71 (0.95)	1 (0.71)	1.1 (-0.08 to 2.2)
Type 4	1.33 (0.58)	1 (NA)		2 (NA)	0 (NA)		2.1 (1.2)	1.14 (0.9)	0.54 (-0.27 to 1.33)	1.22 (0.67)	0.5 (1.07)	0.47 (-0.28 to 1.19)
Type 5	1 (0)	0 (NA)		2 (NA)	0 (NA)		1.78 (0.97)	1.5 (1.64)	0.41 (-0.45 to 1.23)	1.4 (0.89)	0 (0)	1.5 (-0.04 to 2.96)
Type 6	1 (NA)			1.5 (0.58)	0 (0)	2.89 (0.11 to 5.73)	1.83 (1.33)	1 (1.73)	0.26 (-0.92 to 1.39)	1.44 (0.73)	0.43 (0.53)	0.95 (0.02 to 1.84)
Type 7	1 (NA)	0 (NA)					1.2 (0.45)	1 (1.41)	0.15 (-0.85 to 1.12)	1 (NA)	0 (NA)	
Total	1.05 (0.22)	0.14 (0.36)	2.36 (1.31 to 3.39)	1.5 (0.52)	0 (0)	3.06 (1.78 to 4.32)	1.73 (0.95)	1.19 (1.12)	0.34 (0.05 to 0.63)	1.63 (0.91)	0.75 (1.02)	0.69 (0.39 to 0.99)

Note: SI = suicidal ideation; SB = suicidal behavior; BPD = Boderline Personality Disorder; PDD = Persistent Depressive Disorder; ES = Effect size with for change from baseline to post therapy computed as paired Cohen's d estimate within each group; numbers in parentheses represent 95% confidence intervals. Pre and post values are presented as mean (SD). Empty cells for pre and post values represent missing values. Empty cells for ES occur, when ES could not be computed due to zero-variance in the variables.

Supplementary Table 4. Changes in BDI and BSL-23 after CBASP and DBT treatment by CM clusters

		BDI							BSL-23						
		Time				Time x CM cluster			Time				Time x CM cluster		
Diagnosis	CM cluster	Change	Df	F value	P_{FDR}	Df	F value	P_{FDR}	Change	Df	F value	P_{FDR}	Df	F value	P_{FDR}
PDD	Type 1	-3.64	1,130.71	69.76	<.001***	6,134.65	2.82	0.026*	-0.16	1,236	36.78	<.001***	6,236	2.32	0.034*
	Type 2	-9.00							-0.30						
	Type 3	-10.95							-0.35						
	Type 4	-9.19							-0.61						
	Type 5	-13.51							-0.84						
	Type 6	-9.60							-0.29						
	Type 7	-3.67							-0.36						
BPD	Type 1	-9.31	1,161	57.16	<.001***	6,161	0.42	0.868	-0.42	1,160	12.46	0.001**	6,160	0.90	0.493
	Type 2	-11.26							-0.45						
	Type 3	-10.79							-0.46						
	Type 4	-9.62							-0.32						
	Type 5	-12.44							-0.41						
	Type 6	-13.79							-0.56						
	Type 7	-5.39							0.40						

Note: CTQ scores served as the basis for defining clusters of child maltreatment, which were identified through agglomerative hierarchical clustering. CM profiles, labeled types 1–7, are distinguished by varying combinations of maltreatment dimensions; BDI = Beck Depression Inventory; BSL-23 = Borderline Symptom List 23, BPD = borderline personality disorder; PDD = persistent depressive disorder; Df degrees of freedom; Models were adjusted for the baseline severity on the respective scale; P values for model factors were computed using Type III F tests; FDR false-discovery-rate corrected for number of outcomes; * $p < .05$, ** $p < .01$, *** $p < .001$.

Supplementary Table 5. Pairwise comparisons of changes in BDI and BSL-23 after CBASP and DBT treatment between CM clusters derived from the dimensions of the Childhood Trauma Questionnaire

		BDI			BSL-23		
**Diagnosis**	**Contrast**	**Difference in slopes**	**P value**	**$P_{adjusted}$**	**Difference in slopes**	**P**	**$P_{adjusted}$**
PDD	Type 1 - Type 2	5.36 (1.35 to 9.37)	0.009**	0.121	0.14 (-0.14 to 0.41)	0.324	0.955
	Type 1 - Type 3	7.31 (1.87 to 12.75)	0.009**	0.118	0.19 (-0.18 to 0.56)	0.314	0.951
	Type 1 - Type 4	5.55 (0.58 to 10.52)	0.029*	0.298	0.45 (0.11 to 0.78)	0.009**	0.124
	Type 1 - Type 5	9.87 (3.5 to 16.23)	0.003**	0.041*	0.67 (0.24 to 1.1)	0.002**	0.038*
	Type 1 - Type 6	5.95 (0.75 to 11.16)	0.025*	0.271	0.13 (-0.22 to 0.48)	0.465	0.99
	Type 1 - Type 7	0.02 (-9.99 to 10.04)	0.996	1	0.2 (-0.48 to 0.87)	0.564	0.997
	Type 2 - Type 3	1.95 (-3.55 to 7.45)	0.484	0.992	0.05 (-0.32 to 0.42)	0.78	1
	Type 2 - Type 4	0.19 (-4.84 to 5.22)	0.94	1	0.31 (-0.03 to 0.65)	0.072	0.541
	Type 2 - Type 5	4.51 (-1.9 to 10.92)	0.166	0.805	0.54 (0.1 to 0.97)	0.015*	0.185
	Type 2 - Type 6	0.59 (-4.67 to 5.86)	0.824	1	-0.01 (-0.36 to 0.35)	0.976	1
	Type 2 - Type 7	-5.33 (-15.38 to 4.71)	0.295	0.94	0.06 (-0.62 to 0.74)	0.856	1
	Type 3 - Type 4	-1.76 (-8 to 4.47)	0.577	0.998	0.26 (-0.16 to 0.68)	0.226	0.887
	Type 3 - Type 5	2.56 (-4.84 to 9.95)	0.495	0.993	0.48 (-0.01 to 0.98)	0.057	0.471
	Type 3 - Type 6	-1.36 (-7.78 to 5.07)	0.676	1	-0.06 (-0.49 to 0.38)	0.792	1
	Type 3 - Type 7	-7.29 (-17.99 to 3.41)	0.18	0.827	0.01 (-0.71 to 0.73)	0.979	1
	Type 4 - Type 5	4.32 (-2.74 to 11.37)	0.228	0.888	0.23 (-0.25 to 0.7)	0.35	0.966
	Type 4 -	0.4 (-5.63 to	0.895	1	-0.32 (-0.72 to	0.126	0.72

	Type 6	6.43)			0.09)		
	Type 4 - Type 7	-5.53 (-15.99 to 4.94)	0.298	0.942	-0.25 (-0.96 to 0.46)	0.486	0.992
	Type 5 - Type 6	-3.92 (-11.14 to 3.31)	0.285	0.935	-0.54 (-1.03 to - 0.05)	0.03*	0.302
	Type 5 - Type 7	-9.85 (-21.04 to 1.35)	0.084	0.59	-0.48 (-1.23 to 0.28)	0.216	0.875
	Type 6 - Type 7	-5.93 (-16.51 to 4.65)	0.269	0.924	0.07 (-0.65 to 0.78)	0.852	1
BPD	Type 1 - Type 2	1.95 (-5.41 to 9.31)	0.599	0.998	0.03 (-0.46 to 0.51)	0.912	1
	Type 1 - Type 3	1.48 (-6.46 to 9.42)	0.712	1	0.04 (-0.48 to 0.56)	0.887	1
	Type 1 - Type 4	0.32 (-7.95 to 8.59)	0.939	1	-0.11 (-0.65 to 0.43)	0.699	1
	Type 1 - Type 5	3.13 (-4.37 to 10.63)	0.409	0.981	-0.02 (-0.51 to 0.47)	0.946	1
	Type 1 - Type 6	4.48 (-3.82 to 12.78)	0.286	0.934	0.13 (-0.41 to 0.68)	0.627	0.999
	Type 1 - Type 7	-3.92 (-15.91 to 8.07)	0.518	0.995	-0.82 (-1.6 to - 0.04)	0.04*	0.374
	Type 2 - Type 3	-0.47 (-9.5 to 8.55)	0.917	1	0.01 (-0.59 to 0.61)	0.973	1
	Type 2 - Type 4	-1.63 (-10.95 to 7.69)	0.729	1	-0.13 (-0.75 to 0.48)	0.669	1
	Type 2 - Type 5	1.18 (-7.46 to 9.82)	0.787	1	-0.04 (-0.62 to 0.53)	0.879	1
	Type 2 - Type 6	2.53 (-6.82 to 11.87)	0.592	0.998	0.11 (-0.51 to 0.72)	0.733	1
	Type 2 - Type 7	-5.87 (-18.6 to 6.86)	0.362	0.969	-0.85 (-1.68 to - 0.01)	0.047*	0.415
	Type 3 - Type 4	-1.16 (-10.95 to 8.63)	0.814	1	-0.14 (-0.78 to 0.5)	0.659	0.999
	Type 3 - Type 5	1.65 (-7.49 to 10.79)	0.721	1	-0.05 (-0.65 to 0.54)	0.858	1
	Type 3 - Type 6	3 (-6.81 to 12.81)	0.545	0.996	0.1 (-0.54 to 0.74)	0.767	1
	Type 3 -	-5.4 (-18.48 to	0.415	0.982	-0.86 (-1.71 to	0.049*	0.427

	Type 7	7.68)			0)		
	Type 4 - Type 5	2.81 (-6.61 to 12.24)	0.555	0.997	0.09 (-0.53 to 0.7)	0.775	1
	Type 4 - Type 6	4.16 (-5.91 to 14.24)	0.414	0.982	0.24 (-0.42 to 0.9)	0.473	0.991
	Type 4 - Type 7	-4.24 (-17.52 to 9.04)	0.528	0.996	-0.71 (-1.58 to 0.15)	0.105	0.661
	Type 5 - Type 6	1.35 (-8.1 to 10.8)	0.777	1	0.15 (-0.47 to 0.77)	0.631	0.999
	Type 5 - Type 7	-7.05 (-19.86 to 5.76)	0.277	0.929	-0.8 (-1.64 to 0.03)	0.06	0.482
	Type 6 - Type 7	-8.4 (-21.69 to 4.89)	0.213	0.87	-0.95 (-1.82 to -0.08)	0.032*	0.317

Note: CTQ scores served as the basis for defining clusters of child maltreatment, which were identified through agglomerative hierarchical clustering. CM profiles, labeled types 1–7, are distinguished by varying combinations of maltreatment dimensions; BDI = Beck Depression Inventory; BSL-23 = Borderline Symptom List 23, BPD = borderline personality disorder; PDD = persistent depressive disorder; Models were adjusted for the baseline severity on the respective scale; Adjustments for multiple comparisons used tukey HSD correction; * $p < .05$, ** $p < .01$, *** $p < .001$.

Supplementary Table 6. Comparison of predictive accuracy for CM clusters with CTQ total score

Predictor Set	Outcome	Balanced Accuracy	Sensitivity	Specificity	AUC	RMSE	MAE
CTQ sum	Lifetime passive SI	0.51	0.53	0.48	0.53		
CTQ sum	Lifetime unspecific active SI	0.51	0.46	0.56	0.53		
CTQ sum	Lifetime active SI with plan or intent	0.52	0.51	0.53	0.55		
CTQ sum	Lifetime active SI with plan and intent	0.54	0.51	0.57	0.59		
CTQ sum	Lifetime SB without attempt	0.56	0.53	0.60	0.59		
CTQ sum	Lifetime SB with attempt	0.59	0.56	0.63	0.63		
CTQ clusters	Lifetime passive SI	0.51	0.55	0.48	0.50		
CTQ clusters	Lifetime unspecific active SI	0.52	0.56	0.49	0.53		
CTQ clusters	Lifetime active SI with plan or intent	0.55	0.64	0.46	0.56		
CTQ clusters	Lifetime active SI with plan and intent	0.53	0.45	0.61	0.56		
CTQ clusters	Lifetime SB without attempt	0.56	0.46	0.67	0.59		
CTQ clusters	Lifetime SB with attempt	0.58	0.47	0.69	0.60		
CTQ sum	Acute SI at baseline					1.09	0.85
CTQ sum	Acute SI at post measurement					0.98	0.80
CTQ sum	Acute SB at baseline					0.48	0.32
CTQ sum	Acute SB at post measurement					0.20	0.06
CTQ clusters	Acute SI at baseline					1.10	0.86
CTQ clusters	Acute SI at post measurement					0.99	0.79
CTQ clusters	Acute SB at baseline					0.49	0.33
CTQ clusters	Acute SB at post measurement					0.22	0.06

Note: CTQ = Childhood Trauma Questionnaire; AUC = area under the receiver operating characteristic curve; RMSE = root mean squared error; MAE = mean absolute error; SI = suicidal ideations; SB = suicidal behaviors.

Supplementary Figure 1. CONSORT chart of participants

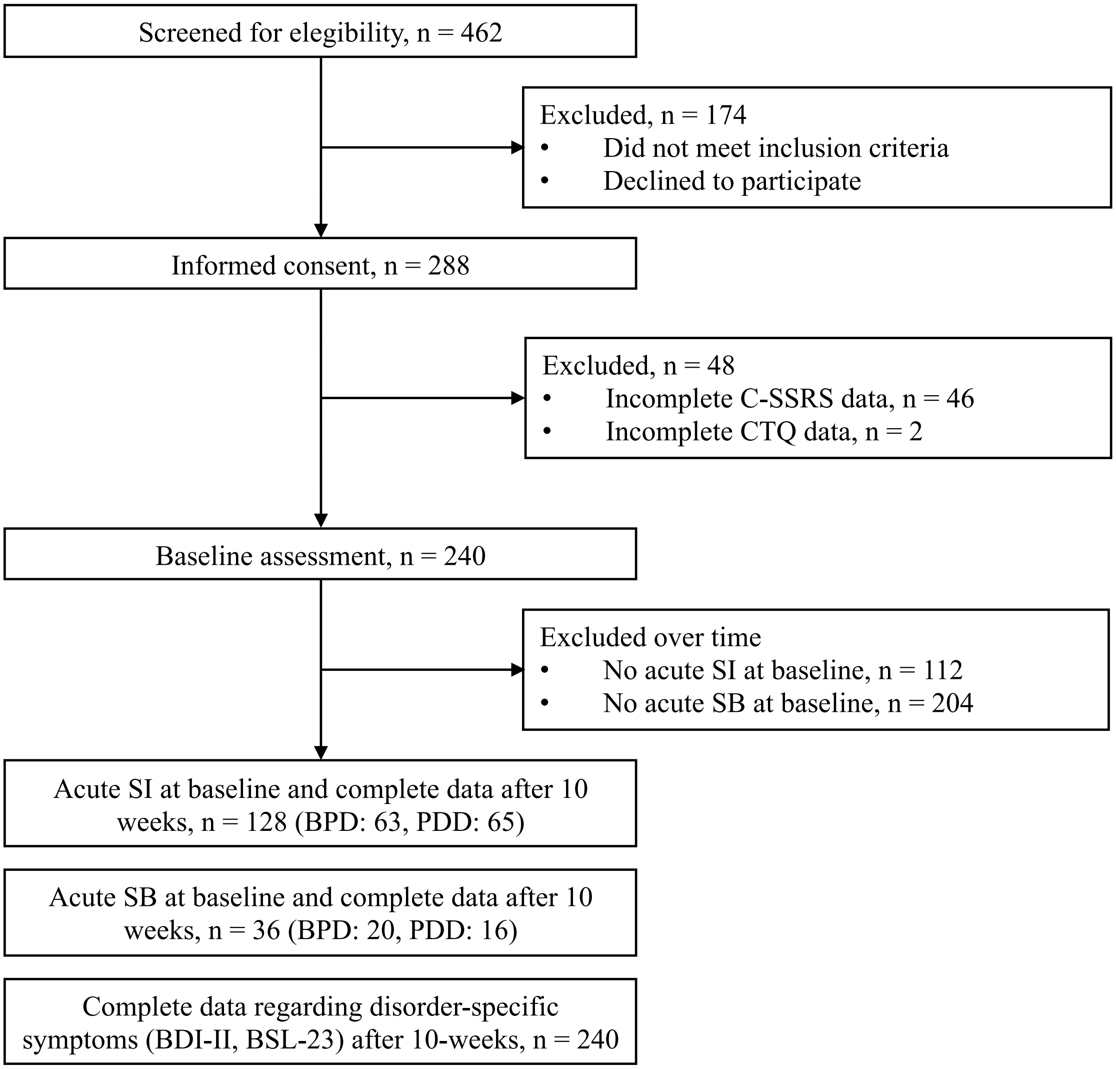

Note. C-SSRS = Columbia-Suicide Severity Rating Scale; CTQ = Childhood Trauma Questionnaire; SI = suicidal ideation; SB = suicidal behavior; BPD = borderline personality disorder; PDD = persistent depressive disorder; BDI-II = Beck Depression Inventopry 2nd Version; BSL-23 = Borderline Sypmtom List.

Supplementary Methods. Description of methods used for comparing predictive utility between clusters and sum scores

To examine the predictive utility of trauma severity versus trauma cluster membership, we compared two single-predictor models across the binary and continuous SIB outcomes. The first predictor was a cumulative CTQ score and the second was CTQ cluster variable. For binary suicidality outcomes, logistic regression models were fitted. To reduce the impact of class imbalance, random undersampling was applied within training folds. Predictive performance was evaluated using repeated 3-fold cross-validation and summarized by balanced accuracy, sensitivity, specificity, and AUC. For continuous symptom outcomes, linear regression models were fitted and evaluated using 10-times repeated 3-fold cross-validation. Predictive accuracy was quantified using RMSE and MAE. All reported results reflect aggregated out-of-sample performance across resampling iterations.
